# Le profil clinique et immunologique de 15 patients Marocains atteints de syndrome hyper IgM

**DOI:** 10.11604/pamj.2017.26.212.10081

**Published:** 2017-04-19

**Authors:** Hind Ouair, Ibtihal Benhsaien, Leila Jeddane, Jalila El Bakkouri, Naima Elhafidi, Noureddine Rada, Jilali Najib, Fatima Ailal, Hanane Salih Alj, Ahmed Aziz Bousfiha

**Affiliations:** 1 Laboratoire de Biologie et Santé, Unité de recherche Associée au CNRST-URAC 34, Faculté des Sciences Ben M'Sik, Université Hassan II Mohammedia, Casablanca, Maroc; 2 Laboratoire d'Immunologie Clinique, Inflammation et Allergie, Faculté de Médecine et Pharmacie, Université Hassan II, Casablanca, Maroc; 3 Département de Pédiatrie 1, Hôpital d'Enfants de Rabat, CHU Ibn Sina, Rabat, Maroc; 4 Département de Pédiatrie, CHU Mohamed VI, Marrakech, Maroc; 5 Département des Maladies Infectieuses, Hôpital A. Harouchi, CHU Ibn Rochd, Casablanca, Maroc

**Keywords:** Syndrome hyper IgM, clinique, immunologie, thérapie, Hyper IgM syndrome, clinic, immunology, therapy

## Abstract

Le Syndrome hyper IgM est un déficit immunitaire héréditaire bien connu, décrit pour la première fois en 1961. Il est causé par un défaut au niveau des lymphocytes B, caractérisé par un taux sérique normal ou élevé des IgM et un taux bas ou nul des IgG, IgA, IgE résultant d'une déficience de la commutation isotypique. Ses manifestations cliniques sont dominées par les infections à répétition, surtout du tube digestif, de la sphère ORL et des poumons. Le syndrome est causé par un défaut de commutation de classe d'immunoglobuline dans les cellules B, et une diminution de la capacité des monocytes à induire la prolifération des lymphocytes T. Le résultat net de tous ces défauts est la susceptibilité accrue aux infections opportunistes à *Pneumocystis jiroveci, Cryptosporidium spp* et d'autres organismes intracellulaires, ainsi qu'une fréquence élevée d'infections bactériennes et virales. L'intérêt de ce projet est d'illustrer l'importance de la compréhension des mécanismes physiopathologiques associés à cette susceptibilité accrue aux infections, ce qui permettra une meilleure prise en charge diagnostique et thérapeutique des patients atteint du Syndrome hyper IgM (SHIM).

## Abstract (en)

Hyper IgM syndrome is a well known genetic (primary) immunodeficiency disorder which was first described in 1961. It is caused by B lymphocyte deficiency characterized by normal or elevated serum IgM levels and low or zero levels of IgG, IgA, IgE resulting from isotype-switching deficiency. Clinical manifestations are dominated by recurrent infections, especially involving the digestive tube of the ENT sphere and the lungs. This syndrome is caused by B-cell immunoglobulin class switch deficiency and decreased capacity to induce proliferation of T lymphocytes. The net result of these deficiencies is reflected in increased susceptibility to Pneumocystis jiroveci, Cryptosporidium spp and other intracellular organisms as well as high rate of bacterial and viral infections. This study aimed to illustrate the importance of understanding the pathophysiological mechanisms associated with this increased susceptibility to infections in order to allow a better diagnosis and therapy in patients with Hyper IgM syndrome (HIM).

## Introduction

Le syndrome hyper IgM (SHIM) ou Class Switch Recombi¬nation Deficiencies (CSR-Ds) constitue un groupe de maladies génétiquement hétérogène. Il est causé par un défaut au niveau de la commutation isotypique en association ou non avec un défaut d'hypermutation somatique dans les lymphocytes B [1]. Les CSR-D sont classés en 6 types, en fonction du gène et de la protéine mutée: SHIM1 (CD40L), SHIM2 (AID), SHIM3 (CD40), SHIM5 (Ung), SHIM6 (NEMO) et SHIM7 (IKBA) [2]. Les manifestations cliniques se développent pendant la première ou la deuxième année de vie pour la majorité des patients et elles sont dominées essentiellement par des infections récurrentes (pulmonaires, ORL, digestives), des infections opportunistes, des signes d'auto-immunité et de lymphoproliferation. Le bilan immunitaire montre un taux bas des IgG, A et E. Une neutropénie d'origine autoimmune peut également être observée.

## Méthodes

Notre étude porte sur 15 cas de CSR-D colligés à l'Unité d'Immunologie Clinique du service de Pédiatrie 1 de l'Hôpital d'Enfants A. Harouchi, CHU Ibn Rochd de Casablanca, depuis Novembre 1995 jusqu'à Décembre 2015. Cette étude a inclus tous les enfants présentant un CSR-D dont l'âge au diagnostic varie de 7 jours à 15 ans. Le diagnostic était suspecté devant une histoire clinique évocatrice de déficit immunitaire, et était confirmé par un bilan immunitaire, comportant une sérologie VIH, le typage des sous populations lymphocytaires (SPL) et le dosage pondéral des Immunoglobulines A, G et M par néphélométrie.

## Résultats

Notre étude a pu colliger 15 patients (7 filles et 8 garçons) atteints de CSR-D (Défaut de Commutation Isotypique) issus de 13 familles différentes, dont 12 cas sont issus d'un mariage consanguin. L'âge d'apparition des premiers signes varie entre 7 jours et 16 mois. L'âge de diagnostic est compris entre 5 mois et 12 ans avec un âge médian de 36 mois. Des infections respiratoires à répétition sont le signe révélateur chez 15 patients, soit 88,8%, compliquées de dilatation de bronches chez 6 cas et de pneumatocèle dans un cas. Le tableau clinique était complété par des infections de la sphère ORL (66,6% des cas), des infections digestives entrainant des diarrhées chroniques (27,7%), un Syndromelymphoprolifératif (11 patients) dont hépatomégalie chez 5 cas, splénomégalie chez 5 cas et adénopathies périphériques dans 6 cas. L'atteinte cutanée est notée chez 5 cas de type abcès de la face, des verrues multiples chez 2 cas, des lésions cutanées érythémato-papuleuses généralisées récidivantes dans 1 cas (Tableau 1). Tous les malades ont eu une sérologie VIH négative. Les SPL sont normales dans tous les cas. Quinze enfants de notre série avaient un taux élevé d'IgM à une moyenne de 3,81 g/L contre 2 malades avec un taux normal. Sur 15 patients marocains cliniquement diagnostiqués comme HIGM, 8 sont encore vivants, sous thérapie de remplacement régulière, associé à une antibioprophylaxie adéquate (Tableau 2).

**Tableau 1:** Description cliniques des patients marocains atteints de Syndrome Hyper IgM

Patient	Sexe	AGE		Histoire Familiale	Consanguinité	Manifestations clinique								Traitement Substitutif	Evolution
		Début de Symptômes (mois)	Diagnostic (mois)			Atteinte respiratoire	RSP	HPM	SPM	ADP	Diarrhées	Muguet buccal	Autres		
N°1:	F	12	36	Non	Oui	+	+	-	-	-	-	+	Lésions cutanées	Non	Perdue de vue
N°2:	F	5	5	Oui	Oui	+	-	+	-	-	+	-	-	Non	Perdue de vue
N°3:	F	67	72	Oui	Oui	+	+	-	-	+	-	-	Sinusite, Rhinite	Oui	Vivant
N°4:	M	36	144	Non	Non	+	+	+	-	-	-	-	Péricardite purulente	Non	Perdu de vue
N°5:	M	8	96	Non	Oui	+	+	+	+	+	+	-	-	Oui	Décédé
N°6:	F	1	7	Oui	Oui	+	-	-	-	-	-	+	Infection à CMV	Oui	Vivant
N°7:	M	6	42	Non	Oui	+	-	-	-	-	-	+	DDB, leishmaniose viscérale	Oui	Vivant
N°8:	M	6	36	Non	Non	+	+	-	+	-	-	-	Otites à répétition	Oui	Décédé
N°9:	M	10	28	Non	Non	-	+	-	-	-	+	-	Cryptosporidiose	Oui	Vivant
N°10:	F	0,2	7	Oui	Oui	+	-	-	+	-	+	+	BCGite, Lésions cutanées	Oui	Décédé
N°11:	F	48	132	Non	Oui	+	-	+	-	+	-	-	Vascularite à ANCA	Oui	Décédé
N°12:	M	6	60	Oui	Oui	+	-		-	+	-	-	Fièvre récurrente, otites	Oui	Vivant
N°13 :	F	6	96	Oui	Oui	+	-	-	-	+	-	-	Verrues multiples, fièvre	Oui	Vivant
N°14:	M	8,5	12	Non	Non	+	+	-	-	-	-	-	-	Oui	Perdu de vue
N°15:	M	8	27	Oui	Oui	+	-	-	-	-	-	-	Abcès du bras, adénite suppurée	Oui	Vivant

RSP: Retard Staturo-Pondérale; HPM: Hépatomégalie; SPM: Splénomégalie ; ADP: Adénopathies ; ANCA: Anticorps Anti-Neutrophiles Cytoplasmique

**Tableau 2:** Bilan immunologique

Observations	Dosage pondéral des Ig (g/L)			Numération des SPL					Etude génétique
	IgM	IgG	IgA	CD3	CD4	CD8	CD19	NK	
N° 1	5.16	2.11	<0.6	1356/ mm^3^	587/ mm^3^	1220/mm^3^	226/ mm^3^	N.D	Séquence AID normale
N° 2	0.58	< 0.5	< 0.13	-	-	-	-	-	-
N° 3	>3.43	< 0.06	< 0.6	2881/ mm^3^	1145/mm^3^	1382/mm^3^	279/ mm^3^	-	Déficit en AID
N° 4	5.16	3.19	< 0.57	-	-	-	-	-	-
N° 5	3.10	2.91	< 0.6	1211/ mm^3^	579/ mm^3^	524/ mm^3^	163/ mm^3^	**1%**	Séquence AID normale Phénotype du lymphocyte B est normal
N° 6	0.967	0.035	0.006	4984/ mm^3^	1299/ mm^3^	2998/ mm^3^	1404/mm^3^	1404/mm^3^	-
N° 7	0.88	< 1.98	< 0.36	4137/ mm^3^	2258/ mm^3^	1513/ mm^3^	1821/mm^3^	188/ mm^3^	Absence d’expression du CD40L
N° 8	2.66	0.940	< 0.01	4900 /mm^3^	1400/mm^3^	2800/mm^3^	900/ mm^3^	130/ mm^3^	-
N° 9	1.16	< 2.11	< 0.37	2600/ mm^3^	1400/ mm^3^	630/ mm^3^	600/ mm^3^	480/ mm^3^	Expression du CD40L sur lymphocyte T = normale Expression du CD40 sur lymphocyte B = normale
N° 10	1.34	< 0.33	< 0.06	2596 /mm^3^	519 /mm^3^	2030/ mm^3^	1520/mm^3^	708/ mm^3^	-
N° 11	9.30	4.72	< 0.05	-	-	-	-	-	-
N° 12	1.95	0.85	0.35	4505 /mm^3^	1872/mm^3^	1930/mm^3^	878/ mm^3^	468/ mm^3^	-
N° 13	2.91	0.80	0.41	1900/ mm^3^	800/ mm^3^	600/ mm^3^	300/ mm^3^	-	-
N° 14	>4.84	<2.11	<0.35	2348/ mm^3^	1057/ mm^3^	1350/ mm^3^	118/mm^3^		-
N° 15	9.33	0.04	0.75	4286/mm^3^	2312/ mm^3^	902/ mm^3^	451/ mm^3^	620/ mm^3^	Déficit en AID

## Discussion

Les syndromes hyper-IgM sont des déficits immunitaires extrêmement rares, décrit pour la première fois en 1961 par Rosen et al d'une façon indépendante [3]. Plusieurs causes moléculaires sont aujourd'hui connues pour expliquer et classer les syndromes HIGM qui peuvent être soit un défaut de signalisation entre les lymphocytes T et B, soit de défauts de transmission intracellulaire du signal induit par l'interaction entre le CD40L et le CD40. Le syndrome hyper IgM lié à l'X représenterait 65 à 70% des syndromes hyper-IgM [4], c'est le plus fréquent en Europe où le taux de consanguinité est très bas et c'est le plus étudié. Le registre américain rapporte que le taux d'incidence minimale de l'X-HIGM était d'environ 1 cas pour 1.030.000 naissances vivantes [5]. Le déficit en AID vient en 2^ème^ position de fréquence après l'X-HIGM. Minegishi a suggéré que 50% des syndromes hyper IgM non lié à l'X étaient des syndromes HIGM2 [6]. Les autres formes, autosomique récessive ou dominante, constituent un groupe hétérogène sur le plan clinique. Aux Etats Unis (2005), dans une large cohorte américaine de 140 patients (dont 130 garçons) ayant un syndrome HIGM, Lee et al rapportent un déficit en CD40L chez 98 des cas (70%), un déficit en AID chez 4 malades (3%), et un défaut du signal NF?B chez un autre malade. Aucune mutation n'a pu être identifiée chez 33 patients (24%) [7]. En Asie, dans une cohorte Iranienne de 33 malades (dont 28 garçons et 5 filles), la consanguinité était notée parmi 9 malades et le profil moléculaire était en faveur des formes autosomiques récessives étant donné le taux important de consanguinité et le phénotype clinique [8]. Alors que dans une série Taïwanaise, Lee (2014) rapporte 14 patients avec un syndrome HIGM appartenant à 12 familles différents dont 10 cas sont identifiés comme formes liées à l'X [9]. Au Maghreb, une étude tunisienne rapportée par Mellouli en 2012 sur 710 cas de DIP colligés entre Avril 1988 et Avril 2012 a montré que les syndromes Hyper IgM représentaient 4,2% des DIP en Tunisie [10]. Sur 502 cas de DIP enregistrés, au Maroc, jusqu'à 2013, les syndromes HIGM représentaient 2.8% des DIP [11]. Une autre étude sur 51 DIP colligés au CHU de Sfax entre 1995 et 2010 a montré que les syndromes Hyper-IgM représentaient 9.8 % de l'ensemble des DIP diagnostiqués [12]. Les patients inclus dans cette étude ont présenté des infections récidivantes caractérisées par leur précocité et gravité. Il s'agit d'infections respiratoires chez 58,8% des cas, dominées par des pneumopathies; des infections du tractus gastro-intestinales révélées par des diarrhées chroniques chez 5 cas (30%); des infections ORL, otites moyennes aigues (30%) et candidose buccale (23,5%).

**Figure 1:**
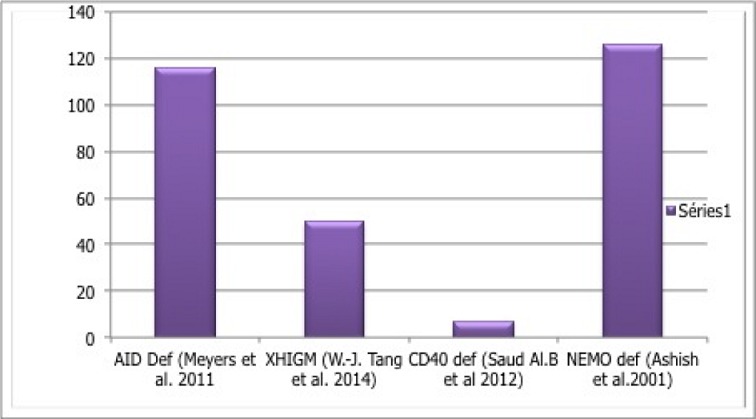
Age médian de diagnostic selon le type des CSR-D

Ainsi, l'âge de diagnostic et les manifestations clinique varient selon le type du SHIM (Tableau 3, Figure 1) [1,13] surviennent au bas âge dès la première année de vie voire dans les premiers mois de vie avec un âge médian de 12 mois [14]. Les patients avec AR-HIGM sont plus âgés au moment du diagnostic que ceux ayant un syndrome HIGM1 ou un syndrome HIGM3. Ainsi pour le syndrome HIGM 2, l'âge est compris entre 1 et 13 ans pour les patients de Revy [13] et entre 2 et 28 ans pour ceux de Minegishi [6]. Dans notre série l'âge médian de diagnostic était de 36 mois. Dans le Registre européen XHIGM, les adénopathies généralisées ont été rapportées dans 7 cas sur 56 patients [15]. L'évolution dans le syndrome HIGM1 est marquée par la répétition des pneumopathies et l'installation de bronchectasies. Un retard de croissance a été rapporté chez certains patients, il est dû essentiellement aux infections à répétition et au retard diagnostique [15]. De plus, les patients avec X-HIGM sont plus fréquemment sujets à développer des Arthrites séronégatives, une encéphalopathie dégénérative, une hypothyroïdie, une néphropathie auto-immune. Les hyperplasies lymphoïdes sont fréquentes dans le syndrome HIGM2: 13 cas sur 18 dans l'étude de Revy, 9 cas sur 18 dans l'étude de Minegishi et les manifestations auto-immunes, (diabète, polyarthrite, hépatite auto-immune, anémie auto-immune, thrombocytopénie, maladie de Crohn, et uvéite chronique) sont observées chez 20 cas sur 29 dans la série de Quartier (Tableau 4) [1, 2, 16, 17]. En dehors des gènes décrits ci-dessus, d'autres défauts moléculaires de la commutation isotypique pouvant conduire à un phénotype HIGM ont été identifiés chez des patients ayant un autre type de DIP avec des mutations des enzymes impliquées dans la réparation de l'ADN: la protéine PMS2, la protéine ATM impliquée dans l'ataxie-télangiectasie [18]. La thérapie de remplacement d'Ig constitue le traitement principal pour corriger les conséquences cliniques du déficit humoralprésent dans toutes les formes du syndrome HIGM. Dans les syndromes HIGM 2, HIGM 4 et HIGM 5, le traitement est basé essentiellement sur les Ig IV, qui réduit sensiblement l'incidence des infections bactériennes. Dans les syndromes HIGM1 et HIGM3, le traitement par les IgIV et l'antibioprophylaxie à base TSU est indiqué chez la majorité des patients en attente de la greffe de CSH, ce qui permet de réduire la fréquence et la gravité des infections mais ne prévient pas la maladie lymphoproliférative, la cholangite sclérosante et les affections malignes [19].

**Tableau 3:** La démographie et description clinique des déficits en CD40

Origine	Sexe	Age début de signes (mois)	Age de diagnostic (mois)	Infections respiratoires à répétition	Pneumonie interstitielle	Diarrhées chroniques	Cholangite chronique	Retard de croissance	Neutropénie	Eosinophilie
Italie	F	24	96	+	+	-	+	-	-	+
^a^ Arabie Saoudite	M	16	60	+	-	-	-	-	+	-
^b^ Arabie Saoudite	F	8	84	+	+	-	-	+	-	+
Turquie	F	10	12	+	+	+	+	+	-	+
Turquie	F	24	32	+	+	-	-	-	+	-
^c^ Turquie	F	36	96	+	+	+	+	-	+	-
^d^ Turquie	M	2	80	+	-	-	-	-	-	-

^+^a et b sont des cousins germains ^+^c et d sont frère et sœur

**Tableau 4:** Age de diagnostic, signes révélateurs et microorganismes dans différentes cohortes sur SHIM

	Maroc (CSRD) n=15	Inde (CD40L def) n = 13	Chine (CD40L def) n = 7	Arabie Saoudite (CD40 def) n= 11	Turquie (AID def) n= 14	Tunisie (AID, CD40L, CD40) n=16
**Age moyen de Diagnostic (mois)**	49	41	49,5	7,63	7,9	60
**Signes révélateurs**	Infections respiratoires, diarrhées chroniques, otites à répétition, muguet buccal	Infections respiratoires, diarrhées chroniques, neutropénie intermittente	Infections respiratoires, diarrhées chroniques, otites, muguet buccal	Infections respiratoires, diarrhées chroniques, Infections ORL,	Infections respiratoires, diarrhées chroniques, Fièvre, Arthrite aseptique, sinusit	Pneumopathie à répétition, Diarrhées chroniques, HSPM, neutropénie, candidose buccale
**Microorganismes identifiés**	Non précis	*Pseudomonas aeruginosa, Cryptosporidium, Klebsiellapneumoniae, Candida, Rotavirus, Candida*	*Enterococcus Faecalis, Cytomegalovirus, Pseudomonas aeruginosa,*Candidaalbicans	*Pneumocystisjirovecipneumonia, Cryptosporidium*	*Haemophilusinfluenzae*	*Staphylococcus albus, Pseudomonas aeruginosa, Streptococcus durans, Proteus mirabilis*

## Conclusion

En conclusion, cette étude a pu rassembler tous les patients HIGM diagnostiqués au sein de l'Unité d'Immunologie clinique, ils présentent des signes cliniques diverses dominés par des Infections respiratoires et des taux normaux ou élevé des IgM.

### Etat des connaissances actuelles sur le sujet

- Le syndrome hyper IgM (SHIM) est une maladie génétiquement hétérogène;
- Il s'agit d'un défaut de la commutation isotypique;
- Se manifeste par des infections récurrentes (pulmonaires, ORL, digestives).

### Contribution de notre étude à la connaissance

- Caractéristiques cliniques des patients marocains pris en charge à l'Hôpital d'enfants AbderahimHarouchi-Casablanca (Centre de Référence des Déficits Immunitaires Primitifs);
- Effet de la consanguinité sur l'évolution de ce syndrome;
- Le suivi et l'évolution de ces patients atteints de syndrome Hyper IgM.
